# Clinical use of [^18^F]fluoro-ethyl-L-tyrosine PET co-registered with MRI for localizing prolactinoma remnants

**DOI:** 10.1007/s11102-024-01430-y

**Published:** 2024-07-23

**Authors:** Victoria R. van Trigt, Leontine E. H. Bakker, Huangling Lu, Iris C. M. Pelsma, Marco J. T. Verstegen, Wouter R. van Furth, Lenka M. Pereira Arias-Bouda, Nienke R. Biermasz

**Affiliations:** 1 Dept. of Medicine, Division of Endocrinology, Center for Endocrine Tumors Leiden, Leiden University Medical Center, Leiden, the Netherlands; 2 Department of Radiology, Section of Nuclear Medicine, Leiden University Medical Center, Leiden, The Netherlands; 3 Department of Neurosurgery, Leiden University Medical Center, Leiden, The Netherlands

**Keywords:** Prolactinoma, Functional imaging, Surgical resection, Disease remission, Adenoma localization, FET, PET

## Abstract

**Purpose:**

To assess the utility of [^18^F]fluoroethyl-L-tyrosine PET co-registered with magnetic resonance imaging ([^18^F]FET-PET/MRI^CR^) in patients with difficult-to-localize prolactinoma to inform clinical decision-making and (surgical) treatment planning.

**Methods:**

Retrospective cohort study of 17 consecutive patients with prolactinoma undergoing [^18^F]FET-PET/MRI^CR^ between October 2020 and September 2022 for either (1) additional information in case of difficult-to-visualize remnants after prior transsphenoidal surgery (TSS), or pharmacological treatment, or (2) radiological diagnosis in absence of a (clear) adenoma on diagnostic/post-treatment conventional MRI.

**Results:**

[^18^F]FET-PET/MRI^CR^ identified a lesion in 14/17 patients, yet failed to identify active lesions in 2 patients with negative conventional MRI despite prolactin > 7.5 times upper limit of normal. [^18^F]FET-PET/MRI^CR^ results were inconclusive in 1 patient due to diffuse tracer uptake 10 weeks post-surgery. [^18^F]FET-PET/MRI^CR^ was completely concordant with a suspected lesion on conventional MRI in 10/17 patients, and partially concordant in 3/17 patients. New foci were identified in 4/17 patients. The [^18^F]FET-PET/MRI^CR^ conclusions influenced clinical shared decision-making in 15/17 patients, of whom 7 patients underwent TSS and 8 refrained from TSS. One patient underwent TSS despite negative [^18^F]FET-PET/MRI^CR^, and one patient underwent additional imaging. Intraoperative findings corresponded with [^18^F]FET-PET/MRI^CR^ in 5/8 patients, and immunohistochemistry was positive in 5/8 patients. The treatment goal was achieved in 7/8 patients, and remission was achieved in 5/7 patients in whom total resection was considered feasible.

**Conclusion:**

[^18^F]FET-PET/MRI^CR^ can be of added value in the preoperative decision-making process for selected patients with difficult-to-localize prolactinoma (remnants), or patients lacking a substrate on conventional MRI.

**Supplementary Information:**

The online version contains supplementary material available at 10.1007/s11102-024-01430-y.

## Introduction

Prolactinomas, albeit rare, are the most common hormone-secreting pituitary adenomas (prevalence 60–100/1,000,000 (United Kingdom)) [1]. Hyperprolactinemia results in various clinical manifestations, including galactorrhea, subfertility, headaches, and psychological complaints. Additionally, large prolactinomas cause mass effects, e.g. visual field defects and neurological deficits [2].

Dopamine agonists (DA), inducing tumor shrinkage and resolution of symptoms, were long regarded as the primary treatment modality, although side effects occur in a subset of patients [3, 4]. Recent guidelines propose consideration of transsphenoidal surgery (TSS) as an alternative first-line treatment modality for small, non-invasive prolactinomas [5], since retrospective observational studies have shown TSS induced higher remission rates and cost-effectiveness, and less side effects than DAs [3, 6, 7].

With the increasing number of requests for prolactinoma surgeries, accurate adenoma localization is important for the selection of prolactinomas feasible for low-risk total resection. While conventional magnetic resonance imaging (MRI) has been the gold standard since the 1990s, localizing small microadenomas and remnants after long-term DA treatment or TSS can be challenging [8]. Consequently, functional imaging, using positron emission tomography (PET), is emerging as an additional diagnostic imaging modality for complex adenomas.

PET has been shown to improve the localization of hormone-producing pituitary adenomas [9–15]. Therefore, a variety of tracers have been developed, including 18-F-fluorodeoxy-glucose ([^18^F]FDG), [^11^C]methionine ([^11^C]MET), and [^18^F]fluoroethyl-L-tyrosine ([^18^F]FET). [^18^F]FDG - the most widely used radiotracer - identified pituitary adenomas with limited sensitivity [16–18], possibly due to its dependency on high metabolic activity, which is typically low in adenomas [19]. More recently, amino acid-based tracers (e.g. [^11^C]MET and [^18^F]FET) were introduced, and used co-registered with MRI ([^11^C]MET-PET/MRI^CR^ and [^18^F]FET-PET/MRI^CR^). [^11^C]MET is a carbon-11-labelled amino acid analog, which is transported into active adenoma tissue via the L-type amino-acid transporter 1 (LAT1). [^11^C]MET-PET/MRI^CR^ is highly effective in localizing pituitary adenomas [9–13, 17], with the highest metabolic activity found in prolactinomas, which positively correlated with serum prolactin levels [17, 20]. The Leiden-Cambridge expert centers’ collaboration resulted in [^11^C]MET-PET/MRI^CR^ being considered useful for decision making for (re)TSS in cases with difficult-to-localize prolactinoma (remnants) [12, 13]. However, the short half-life of [^11^C]MET (i.e. 20 min), necessitates an on-site cyclotron, hampering [^11^C]MET-PET/MRI^CR^ accessibility [21].

[^18^F]FET is an alternative, fluor-18-labelled amino acid analog, transported into the cell via the same LAT1, yet not metabolized nor incorporated into peptides, and widely used in the diagnosis of malignant brain tumors and metastases. [^18^F]FET has the advantage of having a 110-minute half-life, facilitating easier handling and distribution, and potentially improved spatial resolution, due to ^18^F-based tracers having shorter positron ranges [21–23]. A recent preliminary series on 15 patients with Cushing’s Disease showed a 100% positive predictive value for tumor localization using [^18^F]FET-PET/MRI^CR^, and excellent correlation between [^18^F]FET-PET/MRI^CR^ and [^11^C]MET-PET/MRI^CR^ regarding tumor localization (3/3 patients) [24]. In acromegaly, [^18^F]FET-PET/MRI^CR^ proved to be useful in selected cases (*n* = 8) [25]. To date, no studies have reported on the use of [^18^F]FET-PET/MRI^CR^ in patients with prolactinomas. Therefore, the current study aims to describe the results and utiliy of [^18^F]FET-PET/MRI^CR^ for clinical decision-making in a cohort of consecutive patients with difficult-to-localize prolactinomas.

## Methods

### Subjects and clinical care

This cohort study included all patients with prolactinoma with inconclusive adenoma localization on conventional MRI undergoing a [^18^F]FET-PET/MRI^CR^ between October 2020 and September 2022. The need for informed consent for data collection and utilization was waived (G19.011). The decision for use of [^18^F]FET-PET/MRI^CR^ for clinical decision-making and preoperative treatment planning was made by a multidisciplinary team (MDT) on a case-by-case basis and was only performed in patients with a high need for surgical treatment, usually due to side effects of DA treatment. At the time of imaging, all patients had an active prolactinoma, evidenced by elevated prolactin levels (≥ 1.0 times upper limit of normal (xULN)).

All patients were treated by a dedicated MDT at the Pituitary Center of the Leiden University Medical Center (LUMC), a tertiary referral center for pituitary and complex endoscopic skull base surgery, performing approximately 150 surgeries a year.

A predefined Value Based Healthcare care pathway was followed, complying with international guidelines [5, 26], with a comprehensive outcome set being prospectively collected, as described prior [13, 27]. [^11^C]MET-PET/MRI^CR^ was implemented in the LUMC in 2019. In 2020, [^18^F]FET-PET/MRI^CR^ was implemented following government approval, due to logistic issues with [^11^C]MET. The diagnostic clinical protocol was equal to [^11^C]MET [13]. In brief, all patients were discussed in MDT meetings consisting of experienced neurosurgeons, endocrinologists, and neuroradiologists. Functional imaging was performed only if conventional imaging failed to identify (the extension of) the lesion, as functional imaging was considered not cost-effective or necessary in patients with well-defined lesions on conventional MRI [13, 25]. The patient-specific treatment plan was created after careful re-evaluation of all imaging (from diagnosis to the most recent and functional imaging), biochemistry, patient characteristics, prior surgical reports and immunohistochemistry.

### Indication for functional imaging

Patients underwent [^18^F]FET-PET/MRI^CR^ for two indications, as described previously [13]:

1. Imaging performed for *additional information* to determine the extension/invasion of a (remnant) lesion, or possible multifocality in patients with a difficult remnant after TSS and/or longstanding medical treatment with indeterminate findings on conventional MRI.
2. Imaging performed for *radiological diagnosis* in patients with suspected prolactinoma without a (clear) adenoma on conventional MRI at diagnosis, or resulting from TSS and/or medical therapy-induced shrinkage.

### Biochemical and clinical parameters

All study parameters were extracted from the electronic patient records. Serum prolactin levels (reported as ULN) at baseline, i.e. the first known measurement prior to treatment, and the prolactin measurement closest to the date of [^18^F]FET-PET/MRI^CR^ were reported. Disease duration was defined as the time between diagnosis and [^18^F]FET-PET/MRI^CR^. Previous treatment (DA or TSS) was reported, including the duration of DA treatment (< 6 months, 6 months – 1 year, or > 1 year).

Adenoma size was derived from conventional MRI at baseline (first available MRI – before treatment) and at time of [^18^F]FET-PET/MRI^CR^ (no visible adenoma, microadenoma (remnant), macroadenoma (remnant) or giant adenoma (remnant)). Conventional MRIs were assessed both by experienced neuroradiologists and experienced pituitary neurosurgeons. [^18^F]FET-PET/MRI^CR^ results were classified by nuclear radiologists and the MDT as follows: one active lesion concordant with MRI, one active lesion not identified with conventional MRI, multifocal active lesion concordant or discordant with MRI, no active lesion concordant or discordant with MRI, or inconclusive.

The personal treatment proposal based on [^18^F]FET-PET/MRI^CR^ was recorded as used during clinical consultations (i.e. TSS, DA, radiotherapy, or biochemical/radiological surveillance), as well as the final choice of treatment. For patients undergoing TSS, primary surgical goals were either total resection with normalization of prolactin levels, or debulking with clinically relevant tumor reduction, enabling a decrease in DA dose. The likelihood of achieving the primary surgical goal (unlikely:~21–40%, possibly: ~41–60%, likely:~61-~80% or very likely: ~>80%), and risk of complications (low: ~2%, moderate:~2–5% or increased: ~>5%) was estimated by the MDT based on all diagnostic tools and findings upon previous surgeries, as described previously [13]. Histopathological surgical samples were analyzed by experienced clinical pathologists, and were classified as confirmative of prolactinoma if a pituitary adenoma with immunohistochemical staining of prolactin was observed.

Clinically relevant complications were recorded (e.g. syndrome of inappropriate secretion of antidiuretic hormone, meningitis, cerebrospinal fluid leakage, severe epistaxis, new pituitary insufficiency including arginine vasopressin (AVP) deficiency, or any unplanned readmissions), and categorized as either transient (resolving within 6 months), or permanent (persisting ≥ 6 months). Biochemical remission was defined as normalization of prolactin levels (as described above). Clinical remission was defined as near normal prolactin levels (< 2xULN), combined with resolution of symptoms (e.g., restoration of the menstrual cycle and/or resolution of galactorrhea) and no need for treatment [13].

### Immunoassays

Prolactin was measured on a Cobas E602 immuno-analyzer using the Elecsys Prolactin II kit of Roche Diagnostics, Mannheim Germany, with measurement range 0.047–470 ng/ml (or 1.00–10,000 mIU/L). High dose hook effect was not found up to 12,690 ng/ml (270,000 mIU/L). The variation coefficient (VC) was 2.55% at 49.7ng/ml and 2.38% at 5.9 ng/ml, with both values based on 400 + measurements of internal quality control samples.

## Imaging techniques

### Imaging details

#### MRI

MR imaging was performed on an Achieva 3.0 T MR system (Philips Healthcare, Best, The Netherlands) using a commercial 32-channel head coil according to local pituitary protocols, as described earlier [13].

#### PET/computed tomography (CT) imaging with [^18^F]FET ([^18^F]FET-PET/CT)

O-(2-[^18^F]-fluoroethyl)-L-tyrosine was manufactured in compliance with good manufacturing practice at the Radionuclide Centre of the Amsterdam University Medical Center (Amsterdam UMC; location VU University Medical Centre (VUMC), Amsterdam, the Netherlands). DAs were discontinued > 4 weeks prior to the PET. PET-CT scans were acquired at our own institution using a hybrid PET-CT system (Vereos, Philips Healthcare, Best, The Netherlands), according to the European Association of Nuclear Medicine (EANM) guidelines for brain tumor imaging using labelled amino acid analogues [28]. After intravenous injection of 200 MBq [^18^F]FET a low-dose CT of the head (220 mAs, 140 kV, 0.5 s rotation, 0.984 mm pitch, 1 mm slice thickness) was acquired, followed by dynamic PET acquisition of the brain up to 40 min after injection, acquiring 5-min frames.

#### Image processing and co-registration with MRI ([^18^F]FET-PET/MRI^CR^)

Summation images from 10 to 30 min and 20–40 min after injection were co-registered with MRI for clinical reading. Co-registration with MRI was performed using IntelliSpace Portal version 10 (ISP, Philips Healthcare) [13]. Tyrosine uptake maps were individually thresholded to the uptake in the cerebellum (reference tissue). Standardized uptake values (SUV) at the adenoma site and cerebellum were derived and the maximum adenoma-to-background ratio (TBR_max_) was calculated (SUV_max_ adenoma / (SUV_mean_ cerebellum). All co-registered images were reviewed by an experienced nuclear medicine physician and neuroradiologists on dedicated PACS workstations using Sectra IDS7 software.

The MRI images shown in this manuscript were the MRIs performed for co-registration and were therefore not the MRIs performed for indication setting for functional imaging. Notably, the selected MRI images were chosen to show the suspected lesion identified by [^18^F]FET-PET/MRI^CR^, before analysis of functional imaging the interpretation of MRI sequences and sequential scans was therefore more complex.

### Statistics

Data were collected using Castor EDC and data analysis was performed using SPSS for Windows version 29.0 (SPSS Inc., Chicago, IL, USA). Descriptive statistics were performed, and data was presented as number (%), or median (range). Correlation between TBR_max_ and serum prolactin levels at time of [^18^F]FET-PET/MRI^CR^ were assessed using Spearman correlation.

## Results

### General patient characteristics

Seventeen patients (13 (76.5%) females) with prolactinoma were included, of whom clinical characteristics are shown in Table 1. At time of [^18^F]FET-PET/MRI^CR^, median age was 37 (24–59) years, median disease duration was 6 (1–13) years, and median prolactin levels were 3.6 (1.0-20.3)xULN.

At diagnosis, 8/17 patients presented with a microadenoma, and 6/17 patients presented with a macroadenoma, with possible CSI in 2 patients. No certain lesion was visible in 3/17 patients at diagnosis, however a microadenoma developed during the disease course in 2 of them. All patients were pre-treated with DAs, and 9/17 patients had previously undergone TSS.

**Table 1:** Clinical and biochemical data of all patients and per group

	All, *n* = 17	Group 1, *n* = 11 Additional information	Group 2, *n* = 6 Radiological diagnosis
Sex (female)	13 (76.5%)	7 (63.6%)	6 (100%)
Age (years)^a^	37 (24–59)	38 (30–59)	36 (24–49)
Duration of disease (years)^a^	6 (1–13)	7 (3–13)	5.5 (1–12)
Prolactin at diagnosis (xULN)	7.2 (3.3-794.9)	8.3 (4.1-794.9)	4.3 (3.3–15.0)
**MRI at diagnosis**			
No adenoma	3 (17.6%)	2 (18.2%)	1 (16.7%)
Microadenoma	8 (47.1%)	6 (54.5%)	2 (33.3%)
Macroadenoma	6 (35.3%)	3 (27.3%)	3 (50.0%)
Giant adenoma	0 (0.0%)	0 (0.0%)	0 (0.0%)
Cavernous sinus invasion			
*Certain*	0 (0.0%)	0 (0.0%)	0 (0.0%)
*Possible*	2 (11.8%)	2 (18.2%)	0 (0.0%)
*Unknown*	2 (11.8%)	0 (0.0%)	2 (33.3%)
**Prior treatment**			
Surgery	9 (52.9%)	6 (54.5%)	3 (50.0%)
Medication (DA)	17 (100.0%)	11 (100.0%)	6 (100.0%)
**Duration of medical treatment** ^**b**^			
< 6 months	3 (17.6%)	2 (18.2%)	1 (16.7%)
6 months – 1 year	0 (0.0%)	0 (0.0%)	0 (0.0%)
> 1 year	11 (64.7%)	6 (54.5%)	5 (83.3%)
**Hormone levels at time of [** ^**18**^ **F]FET-PET/MRI** ^**CR**^			
Prolactin at [^18^F]FET-PET/MRI^CR^ (xULN)^c^	3.6 (1.0-20.3)	3.4 (1.0-20.3)	5.5 (2.1–10.8)
**MRI before [** ^**18**^ **F]FET-PET/MRI** ^**CR**^			
Macroadenoma remnant	1 (5.9%)	1 (9.1%)	0 (0.0%)
Microadenoma remnant	2 (11.8%)	2 (18.2%)	0 (0.0%)
No remnant	6 (35.3%)	0 (0.0%)	6 (100.0%)
Possible adenoma remnant	4 (23.5%)	4 (36.4%)	0 (0.0%)
Possible multifocal remnant	4 (23.5%)	4 (36.4%)	0 (0.0%)
**[** ^**18**^ **F] FET-PET/MRI** ^**CR**^			
One active lesion concordant with MRI^d^	9 (52.3%)	9 (81.8%)	0 (0.0%)
One active lesion not identified with MRI	4 (23.5%)	0 (0.0%)	4 (66.7%)
No active lesion	3 (17.6%)	1 (9.1%)^e^	2 (33.3%)
Multifocal active lesion	1 (5.9%)	1 (9.1%)	0 (0.0%)
**Treatment advice based on [** ^**18**^ **F]FET-PET/MRI** ^**CR**^			
Surgery offered	11 (64.7%)	8 (72.7%)	3 (50.0%)
No surgery	6 (35.3%)	3 (27.3%)	3 (50.0%)

Values are presented as median (range), or number (percentage)

*DA* dopamine agonist, x*ULN* times upper limit of normal

^a^ At time of [^18^F]FET-PET/MRI^CR^

^b^ Missing data, *n* = 3

^c^ Dopamine agonists were discontinued > 4 weeks before [^18^F]FET-PET/MRI^CR^ in all patients

^d^ The lesion on [^18^F]FET-PET/MRI^CR^ corresponded with one out of two possible lesions in the patients with suspicion of a multifocal lesion on conventional MRI (*n* = 2)

^e^ Inconclusive result due to diffuse tracer uptake in the sellar region

### [^18^F]FET-PET/MRI^CR^ indications

Supplementary Table 1 shows a detailed overview of tumor characteristics and treatment outcomes per patient.

Eleven patients underwent [^18^F]FET-PET/MRI^CR^ for additional information (group 1). Additional information was required due to possible multifocality in 4 patients, and to determine the exact localization and parasellar extension of remnants in 7 patients (postoperatively in 5 patients, and after DA treatment in 2 patients).

Six patients underwent [^18^F]FET-PET/MRI^CR^ for diagnosis, as no certain lesion was present on conventional MRI at time of [^18^F]FET-PET/MRI^CR^ (group 2).

### [^18^F]FET-PET/MRI^CR^ results

**Fig. 1:**
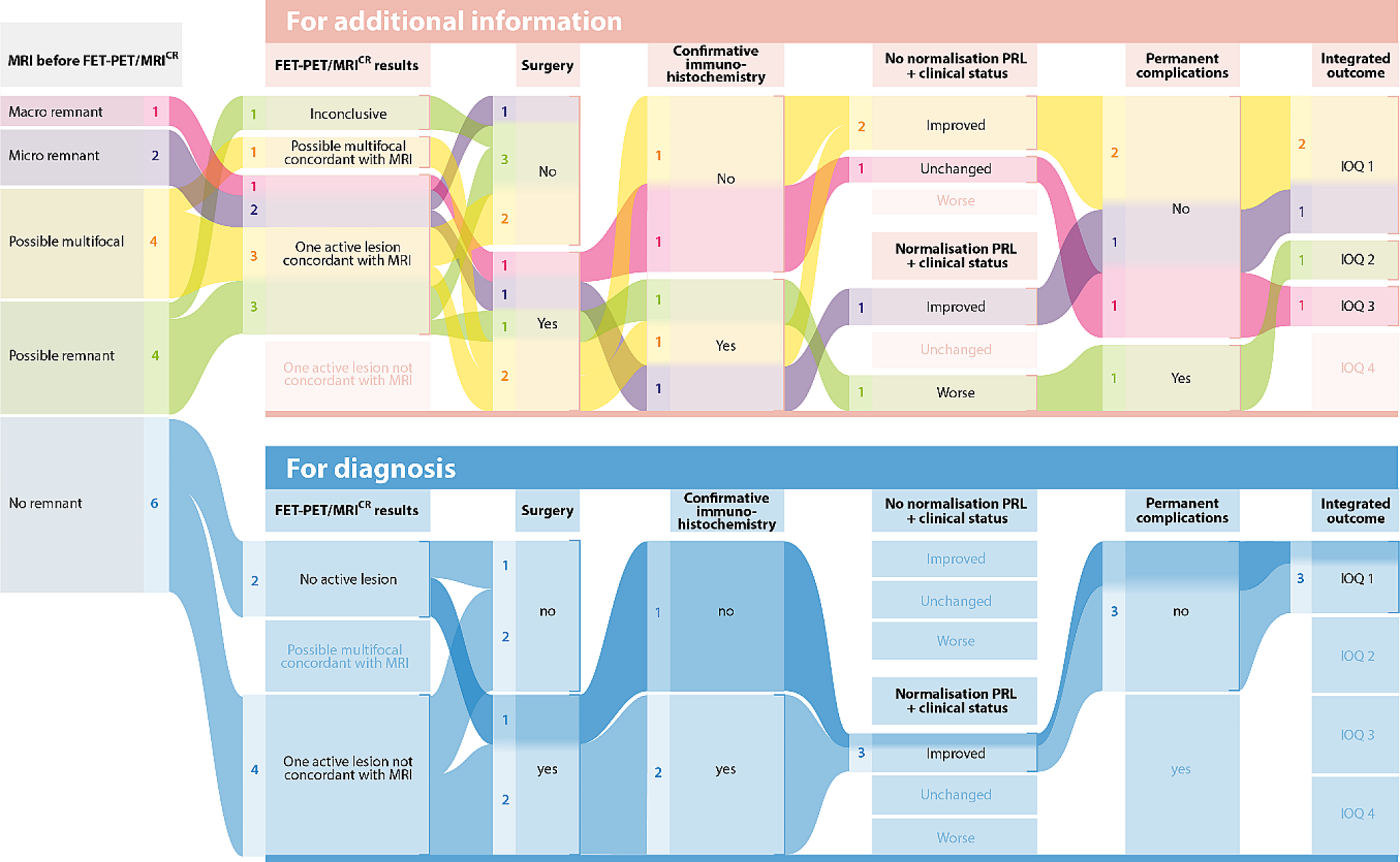
Flow chart of clinical and radiological outcomes for all patients. Sanky diagram of patients undergoing [^18^F]FET-PET/MRI^CR^. Values are presented as individual patients belonging to the described diagnostic group. *IOQ* integrated outcome quadrants: *IOQ 1* – treatment goal achieved without permanent complications, *IOQ 2* – treatment goal achieved with permanent complication, *IOQ 3* – treatment goal not achieved, without permanent complications, *IOQ 4* – treatment goal not achieved with permanent complications, *PRL* prolactin

**Fig. 2:**
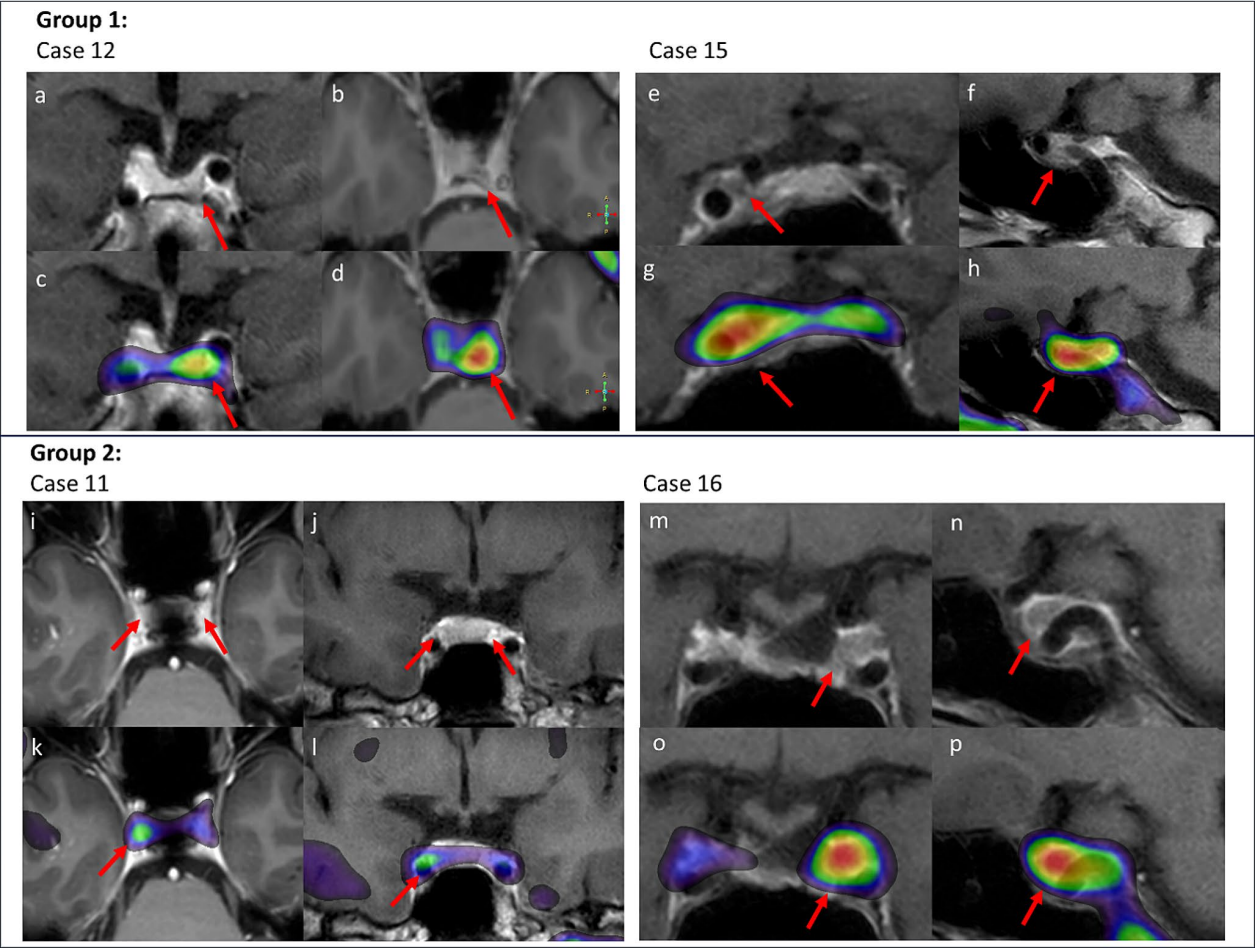
[^18^F] FET-PET/MRI^CR^ images. Four illustrative patients who underwent [^18^F]FET-PET/MRI^CR^ are described in detail. **Group 1: *Case 12***: Female patient who experienced a recurrence two years post-surgery. She underwent [^18^F]FET-PET/MRI^CR^ to assess the extension of the lesion. (a) Coronal T_1w_ MRI (post-contrast) with the suspected lesion left posterior in the sella, (b) axial T_1w_ MRI (post-contrast) (c) [^18^F]FET-PET/MRI^CR^ fusion (coronal) showed increased focal activity corresponding to conventional MRI, (d) [^18^F]FET-PET/MRI^CR^ fusion (axial). ***Case 15***: Male patient with persistent disease post-surgery undergoing [^18^F]FET-PET/MRI^CR^ to determine the extension of the lesion for assessment of surgical possibilities. (e) Coronal T_1w_ MRI (post-contrast) with a suspected residue right sellar, (f) Sagittal T_1w_ MRI (post-contrast), (g) [^18^F]FET-PET/MRI^CR^ fusion (coronal) showed increased focal uptake right posteriorly in the suspected lesion, (h) [^18^F]FET-PET/MRI^CR^ fusion (sagittal). **Group 2: *Case 11***: Female patient treated with cabergoline for two years. [^18^F]FET-PET/MRI^CR^ was performed for radiological diagnosis of an adenoma. (i) Axial T_1w_ MRI (post-contrast) with two dubious lesions on both sides in the sella region, yet no certain adenoma (j) coronal T_1w_ MRI (dynamic contrast enhanced) showed the two dubious lesions on both sides without enhancement, (k) [^18^F]FET-PET/MRI^CR^ fusion (axial) showed increased focal activity right posterior in the sella. There was no increased activity on the left side, (l) [^18^F]FET-PET/MRI^CR^ fusion (coronal) showed the increased focal activity in the dubious lesion on the right side. ***Case 16***: Female patient underwent [^18^F]FET-PET/MRI^CR^ to determine the location of the prolactinoma remnant as a target for radiotherapy due to persistent disease after two prior surgeries. The MRI used for indication setting (not shown in manuscript) showed an area of reduced enhancement on the left, yet it was unsure whether this concerned a resection cavity or residual adenoma. (m) Coronal T_1w_ MRI (post-contrast) showed either residual adenoma or postoperative changes left in the cavernous sinus (n) Sagittal T_1w_ MRI (post-contrast), (o) [^18^F]FET-PET/MRI^CR^ fusion (coronal) with increased focal uptake left anterior of the carotid artery in the location of the lesion on MRI, (p) [^18^F]FET-PET/MRI^CR^ fusion (sagittal)

[^18^F]FET-PET/MRI^CR^ identified a single lesion in 13/17 patients, multifocal active lesions in 1/17 patients, no clear lesion in 2/17 patients, and was inconclusive in 1/17 patients. In group 1, positive [^18^F]FET-PET/MRI^CR^ uptake was completely concordant with a suggestive MRI lesion in 7/11, and partially concordant in 3/11 patients – showing one active lesion on [^18^F]FET-PET/MRI^CR^ and possible multifocality on MRI. [^18^F]FET-PET/MRI^CR^ was inconclusive in one patient of group 1 (#10). In group 2, 4/6 patients had an active lesion on [^18^F]FET-PET/MRI^CR^ despite a negative conventional MRI. [^18^F]FET-PET/MRI^CR^ was negative in 2/6 patients of group 2 (#7, 9), being concordant with a negative conventional MRI. A flow chart of radiological and clinical outcomes is depicted in Fig. 1, and imaging for 4 exemplary cases is shown in Fig. 2.

### [^18^F]FET-PET/MRI^CR^-guided personal treatment advice

Based on [^18^F]FET-PET/MRI^CR^, TSS aiming for total resection was offered to 11/17 patients (3/11 *likely* chance of total resection, 6/10 *possible* chance, and 2/10 *unlikely* chance). In patient #15, the *unlikely* chance was due to the adenoma’s localization between the bifurcation of the carotid artery with possible CSI, yet she was offered surgery due to a high need for alternative treatment. A low-risk surgical attempt with an *unlikely* chance of total resection was agreed upon in patient #9 (group 2) with negative conventional MRI and [^18^F]FET-PET/MRI^CR^ because of a high disease burden and severe intolerance for DAs (prolactin 10.8xULN). Surgery aiming at debulking was proposed in one patient of group 1, with an estimated *possible* chance of achieving the goal.

The MDT advised against surgery in 5/17 patients, since total resection was considered *unlikely* due to the lesion’s localization, without expected benefit of debulking in 3 patients, and due to negative [^18^F]FET-PET/MRI^CR^ in 1 patient (despite of prolactin 7.5xULN and discontinuation of DA > 9 weeks prior to scan). In patient #10, [^18^F]FET-PET/MRI^CR^ was performed 10 weeks postoperatively, showing diffuse, moderately increased sellar tracer uptake, possibly due to postoperative mucosal inflammation, which led to repeating functioning imaging (with [^11^C]MET) at a later stage (see below).

### Surgical confirmation of [^18^F]FET-PET/MRI^CR^ and outcomes

An overview of surgical outcomes is shown in Table 2. TSS was performed in 8/17 patients. Surgical intervention confirmed the [^18^F]FET-PET/MRI^CR^ findings in 7/8 patients: by clear intraoperative lesions and positive immunohistochemistry in 5/8 patients, and clinically relevant prolactin decrease in 7/8 patients. Histopathology was indicative of chronic hypophysitis rather than an adenoma in patient #9.

**Table 2:** Treatment outcomes of surgically treated patients for all patients and per group

	All, *n* = 8	Group 1, *n* = 5 Additional information	Group 2, *n* = 3 Radiological diagnosis
Confirmative immunohistochemistry	5 (62.5%)	3 (60.0%)	2 (66.7%)
Normalization prolactin	5 (62.5%)	2 (40.0%)	3 (100.0%)
IOQ			
*1*	6 (75.0%)	3 (60.0%)	3 (100.0%)
*2*	1 (12.5%)	1 (20.0%)	0 (0.0%)
*3*	1 (12.5%)	1 (20.0%)	0 (0.0%)
*4*	0 (0.0%)	0 (0.0%)	0 (0.0%)
Clinical status			
*Improved*	6 (75.0%)	3 (60.0%)	3 (100.0%)
*Unchanged*	1 (12.5%)	1 (20.0%)	0 (0.0%)
*Worse*	1 (12.5%)	1 (20.0%)	0 (0.0%)
Complications	2 (25.0%)	1 (20.0%)	1 (33.3%)
*Transient*	1 (12.5%)	0 (0.0%)	1 (33.3%)
*Permanent*	1 (12.5%)	1 (20.0%)	0 (0.0%)

Values are presented as median (range), or number (percentages)

*IOQ* integrated outcome quadrant: *IOQ 1* – treatment goal achieved without permanent complications, *IOQ 2* – treatment goal achieved with permanent complication, *IOQ 3* – treatment goal not achieved, without permanent complications, *IOQ 4* – treatment goal not achieved with permanent complications

The surgical goal was achieved in 7/8 patients, with normalized prolactin in 6 patients, and clinically irrelevant marginally elevated prolactin levels (1.1-1.6xULN), but symptom resolution and restoration of menstrual cycle in one patient. A permanent complication occurred only in one patient (PTSD in patient already having an anxiety disorder). In patient #15, for whom the chance of total resection was estimated *unlikely*, the goal was indeed not achieved, nor was any clinical improvement.

The other eight patients did not undergo surgery after [^18^F]FET-PET/MRI^CR^, because of either the low likelihood of total resection based on functional imaging (*n* = 4), the mildness of symptoms and/or uncertainty about the symptoms being caused by the prolactinoma (*n* = 3), or postponement of surgery due to personal reasons (*n* = 1).

### SUV ratios and prolactin levels

No correlation was found between TBR_max_ and the prolactin levels at time of [^18^F]FET-PET/MRI^CR^ (*p* = 0.776, *r* = 0.084).

### [^18^F]FET-PET/MRI^CR^ vs. [^11^C]MET-PET/MRI^CR^

**Fig. 3:**
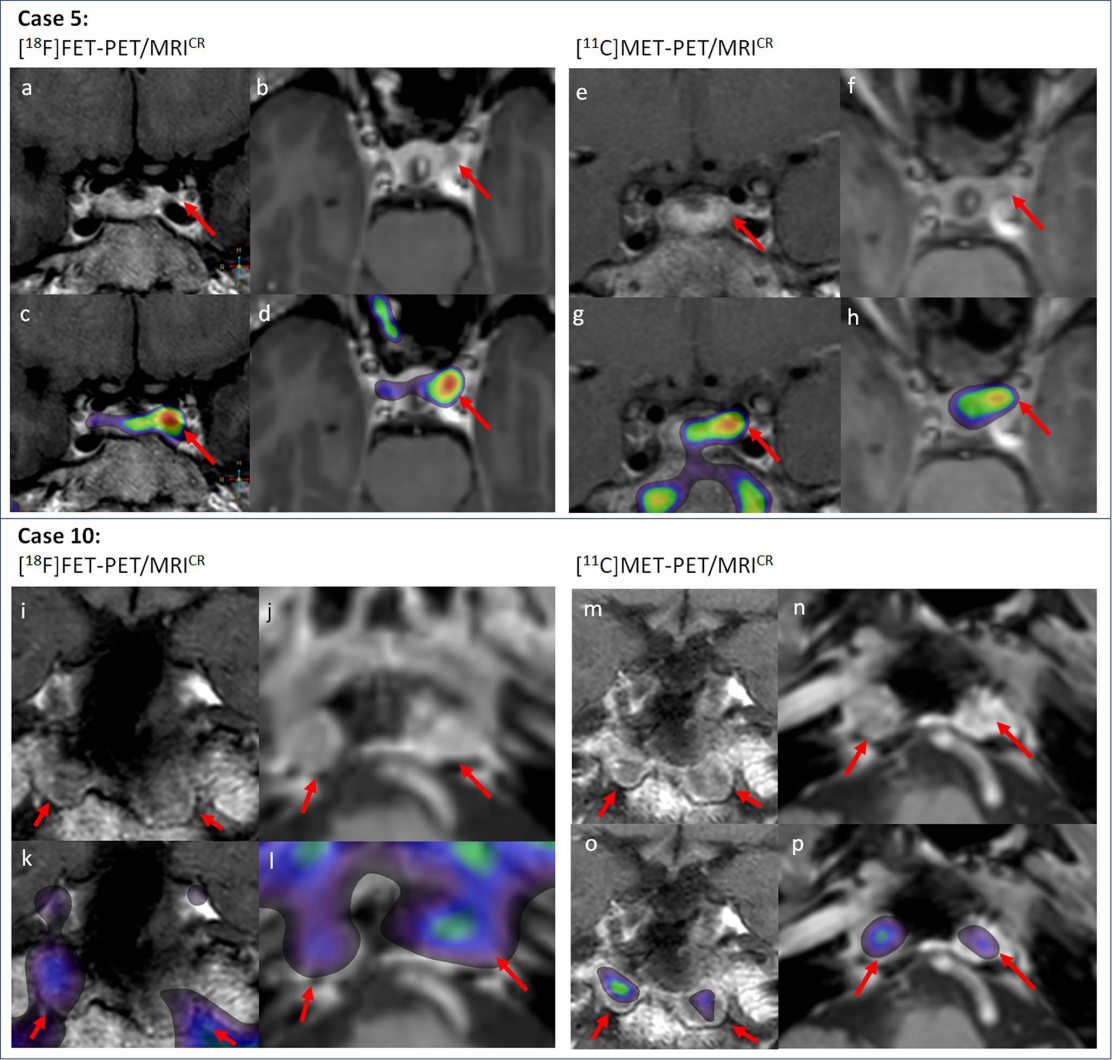
[^18^F] FET-PET/MRI^CR^ and [^11^C]MET-PET/MRI^CR^ images. **Case 5:** Female patient who underwent [^11^C]MET-PET/MRI^CR^ due to persistent disease post-surgery (2019). (e) Coronal T_1w_ MRI (post-contrast) with the suspected residual adenoma left anterolateral in the sella with extension in the cavernous sinus (f) axial T_1w_ MRI (post-contrast) showed the suspected lesion left anterolateral in the sella, (g) [^11^C]MET-PET/MRI^CR^ fusion (coronal) showed increased activity in the suspected lesion, (h) [^11^C]MET-PET/MRI^CR^ fusion (axial). Following this imaging biochemical surveillance was continued due to the mildness of symptoms and a low likelihood of total resection. In 2021 new irregularity of the patient’s menstrual cycle necessitated reevaluation of treatment options. Sequential MRIs raised the suspicion of multifocality, for which [^18^F]FET-PET/MRI^CR^ was performed (a) Coronal T_1w_ MRI (post-contrast) with suspected residual adenoma left anterolateral in the sella with extension in the cavernous sinus, (b) axial T_1w_ MRI (post-contrast) showed the suspected lesion left anterolateral in the sella, (c) [^18^F]FET-PET/MRI^CR^ fusion (coronal) confirms the location of the suspected lesion with increased focal activity left anterolateral in the sella, (d) [^18^F]FET-PET/MRI^CR^ fusion (axial). ***Case 10***: Male patient with residual disease after two surgeries. [^18^F]FET-PET/MRI^CR^ was performed to identify the most active location of residual tissue for future treatment (TSS or radiotherapy). (i) Coronal T_1w_ MRI (post-contrast) with two possible locations in the sella floor with extension into the destructed clivus, (j) axial T_1w_ MRI (post-contrast), (k) [^18^F]FET-PET/MRI^CR^ fusion (coronal) showed diffuse moderate activity on both sides in the sella floor, possible locations of residual adenoma, yet inconclusive due to diffuse tracer uptake (10 weeks postoperative), (l) [^18^F]FET-PET/MRI^CR^ fusion (axial). Nine months later [^11^C]MET-PET/MRI^CR^ was performed for confirmation. (m) Coronal T_1w_ MRI (post-contrast) showed the two possible locations adjacent to the sphenoid sinus with extension into the destructed clivus, (n) axial T_1w_ MRI (post-contrast), (o) [^11^C]MET-PET/MRI^CR^ fusion (coronal) with clearly increased focal uptake in the suspected locations, on the right more than on the left side (p) [^11^C]MET-PET/MRI^CR^ fusion (axial).

Two patients underwent both [^11^C]MET-PET/MRI ^CR^ and [^18^F]FET-PET/MRI^CR^, of whom imaging is shown in Fig. 3. In patient #5, [^18^F]FET-PET/MRI^CR^ – performed to assess surgical possibilities upon new irregularity of the patient’s menstrual cycle – corresponded with [^11^C]MET-PET/MRI^CR^ performed two years prior (no treatment took place between the scans), showing increased tracer uptake left anterolateral in the sella, reaching between the bifurcation of the carotid artery. In patient #10, [^18^F]FET-PET/MRI^CR^ – performed 10 weeks postoperatively – showed suspicious bilateral petroclival uptake, although the images were difficult to interpret due to interfering activity in the SC and diffuse and moderately increased uptake in the sphenoid mucosa. Nine months postoperatively, [^11^C]MET-PET/MRI^CR^ identified two lesions (petroclival right and left), which have been successfully debulked. In retrospect, [^11^C]MET-PET/MRI^CR^ uptake pattern corresponded with [^18^F]FET-PET/MRI^CR^ uptake pattern, and the uptake in the sphenoid sinus seen on [^18^F]FET-PET/MRI^CR^ was hypothesized to be caused by postoperative mucosal inflammation.

## Discussion

The present study describes our approach to diagnosis and treatment of complex prolactinoma cases using [^18^F]FET-PET/MRI^CR^, demonstrating its added value for identification of remnants and thereby aiding in shared decision-making and surgical planning in selected patients with prolactinoma. [^18^F]FET-PET/MRI^CR^ was able to identify lesions as potential targets for TSS in patients in whom conventional MRI failed to localize (the extension of) the lesion, and aided in assessment of the likelihood of total resection.

Recently, the landscape of prolactinoma treatment has changed, from DAs being the primary treatment to TSS being considered a viable first-line alternative in non-invasive tumors [3, 5–7, 29]. Optimal visualization of tumor (remnants) is key for selecting surgical candidates. In the setting of an experienced team, functional imaging has added value for complex patients, in whom the lesion is not readily detectable using conventional MRI [9, 12, 13, 17, 20, 30]. [^11^C]MET-PET/MRI^CR^ is the technique most frequently used, whereas [^18^F]FET-PET/MRI^CR^ is an attractive alternative since no on-site cyclotron is required. Yet, limited data on [^18^F]FET-PET/MRI^CR^ for this indication are reported in literature [24, 25].

The purpose of the present study was therefore to determine if [^18^F]FET-PET/MRI^CR^ is useful in the management of complex prolactinoma cases. [^18^F]FET-PET/MRI^CR^ was performed for two indications: (1) for additional information on a potential target lesion, or (2) for radiological diagnosis when conventional MRI failed to identify a lesion. [^18^F]FET-PET/MRI^CR^ identified a positive lesion in 82% of patients, among which four out of six patients with negative conventional MRIs. Moreover, [^18^F]FET-PET/MRI^CR^ results influenced clinical decision-making in 88% of cases. Eight patients underwent TSS, confirming [^18^F]FET-PET/MRI^CR^ findings, and achieving the surgical goal in 86%. [^18^F]FET-PET/MRI^CR^ was inconclusive due to postoperative inflammation in one patient (#10), and failed to identify a lesion in two patients (#7, 9). Thus, [^18^F]FET-PET/MRI^CR^ was useful in most cases, aiding in clinical decision-making.

To create optimal circumstances for adenoma localization, DAs were withdrawn in advance. Based on the half-life of cabergoline, a withdrawal period of > 4 weeks was previously recommended [13]. No previous recommendations were made for quinagolide or bromocriptine. However, a period of > 1 week is advisable – based on their half-life and biological properties – provided the prolactin level has increased sufficiently (≥ 2xULN) after withdrawal. The required withdrawal period may, however vary based on the duration and dose of DA treatment, with highly responsive tumors that have shrunk considerably requiring a longer period. Furthermore, timing of postoperative functional imaging is complex. In the absence of evidence, we would advise waiting for ≥ 3 months if clinically feasible, as diffusely increased uptake in the sphenoid region was observed ten weeks postoperatively, impacting interpretability. Generally, clinical circumstances should be optimized to improve chances of identifying an active lesion.

Several remarkable observations need addressing. Firstly, even patients with only subtle prolactin level elevations (i.e. 1.0-2.5xULN) showed [^18^F]FET uptake, and this technique, therefore, seems to be highly sensitive for intrasellar lesions. Secondly, one patient with a negative [^18^F]FET-PET/MRI^CR^ showed signs of a chronic hypophysitis upon histopathological evaluation rather than prolactinoma tissue. Therefore, the preoperative diagnosis can be questioned in this patient. Thirdly, even though [^18^F]FET-PET/MRI^CR^ was performed in only those patients in whom TSS was seriously considered, 3 patients decided not to proceed with TSS after an operable lesion was identified. Hence, an algorithm needs to be developed to prevent unnecessary functional imaging, which is time-consuming and increases costs. We believe an experienced MDT overseeing the full trajectory is highly important for careful repeated counseling, and optimal use of this functional imaging modality.

In the present study, [^18^F]FET-PET/MRI^CR^ and [^11^C]MET-PET/MRI^CR^ showed good correspondence in uptake pattern in the two patients undergoing both examinations. Comparing the [^18^F]FET and [^11^C]MET tracers, a clear benefit of [^18^F]FET is its longer half-life, enabling its use in centers lacking a cyclotron. Moreover, [^18^F]FET-PET/MRI^CR^ may be more sensitive for small intrasellar lesions with low metabolic activity, as the normal pituitary tissue takes up less [^18^F]FET compared to [^11^C]MET [25]. However tracer wash out in the CS, as was observed in acromegaly [25], may hamper the interpretation of [^18^F]FET-PET/MRI^CR^ in cases with parasellar extension – particularly when the uptake is moderate (notably not worsening outcomes in the present study). While awaiting larger cohorts assessing the sensitivity of [^18^F]FET-PET/MRI^CR^ in the parasellar region, [^18^F]FET-PET/MRI^CR^ should be used cautiously in cases with possible CS involvement, especially in centers with limited experience with functional imaging.

[^18^F]FET-PET/MRI^CR^ uptake ratios – indicative of metabolic activity of the lesions – were not correlated to serum prolactin levels. For comparison, [^11^C]MET-PET/MRI^CR^ uptake ratios were analyzed in our recently published cohort of patients with a prolactinoma, showing no correlation to serum prolactin levels either (*p* = 0.443, *r* = 0.193) [13]. These findings opposed earlier studies [17, 20], which reported a linear relationship between methionine uptake and prolactin levels, although the statistical methods used were not reported. The present findings may be explained by the fact that our cohort consisted of complex cases with pretreated remnants. Additionally, in case of [^18^F]FET-PET/MRI^CR^, wash out of tyrosine may play a role. Dynamic studies in larger populations with treatment-naïve adenomas should be performed for more adequate analysis of the relationship between tyrosine uptake and prolactin levels.

In this small study in which we share the results of complex cases involving [^18^F]FET-PET/MRI^CR^, descriptives rather than predictive statistics were used. Not all [^18^F]FET-PET/MRI^CR^ results could be verified, as not all patients underwent TSS. Furthermore, it should be stressed that not all conventional MRIs preceding functional imaging were completely negative, yet were inconclusive due to uncertainty about the localization or extension of the lesion in patients who were pretreated. The discovery rates of [^18^F]FET-PET/MRI^CR^ in patients with a negative conventional MRI may therefore be lower.

In conclusion, [^18^F]FET-PET/MRI^CR^ may be of added value for assessment of prolactinoma remnants and their extensions, or to localize prolactinomas unidentifiable with conventional MRI, particularly when no on-site cyclotron is available.

## Electronic supplementary material

Below is the link to the electronic supplementary material.

Supplementary Material 1

## Data Availability

No datasets were generated or analysed during the current study.
