# Supplementary material for: Clinical use of [18F]fluoro-ethyl-L-tyrosine PET co-registered with MRI for localizing prolactinoma remnants
Source: Pituitary. 2024 Jul 23;27(5):614–24. doi: 10.1007/s11102-024-01430-y (PMC11513721; doi:10.1007/s11102-024-01430-y)
Supplement: Supplementary file 1 — Supplementary Material 1 [file 11102_2024_1430_MOESM1_ESM.docx]

# Clinical use of [18F]fluoro-ethyl-L-tyrosine PET co-registered with MRI for localizing prolactinoma remnants

Victoria R. van Trigt^1^, Leontine E.H. Bakker^1^, Huangling Lu^2^, Iris C.M. Pelsma^1^, Marco J.T. Verstegen^3^, Wouter R. van Furth^3^, Lenka M. Pereira Arias-Bouda^2^ ***‡***, Nienke R. Biermasz^1^ ***‡***

### Supplemental files

### Author affiliations:

1. Dept. of Medicine, Division of Endocrinology, and Center for Endocrine Tumors Leiden, Leiden University Medical Center, Leiden, the Netherlands
2. Department of Radiology, Section of Nuclear Medicine, Leiden University Medical Center, Leiden, The Netherlands
3. Department of Neurosurgery, Leiden University Medical Center, Leiden, The Netherlands

*‡ Shared last author.*

### Corresponding author:

V. R. van Trigt

Email: v.r.van_trigt@lumc.nl

**Supplementary Table 1** overview of patient and tumor characteristics per patient

| **ID** | **Age (y), sex** | **Baseline** | | **Prior treatment** | | **At time of [^18^F]FET-PET/MRI^CR^** | | | | **MDT estimation of chance to achieve surgical goal** | **Treatment after FET-PET/MRI^CR^** | **intraoperative findings correspond with [^18^F]FET-PET/MRI^CR^/ IHC** | **Complications** | **Biochemical remission** | **Clinical status** | **IOQ ^a^** |
| --- | --- | --- | --- | --- | --- | --- | --- | --- | --- | --- | --- | --- | --- | --- | --- | --- |
|  |  | **PRL (xULN)** | **MRI** | **Type** | **DA**  **Duration** | **PRL (xULN)** | **MRI** | **[^18^F]FET-PET/MRI^CR^** | **TBRmax** |  |  |  |  |  |  |  |
| **Group 1: [^18^F]FET-PET/MRI^CR^ for additional information** | | | | | | | | | | | | | | | | |
| 2 | 57, M | 4.3 | No certain adenoma | DA | >1 y | 8.4 | Possible multifocality | Active lesion left, CSI- | 2.61 | Likely (TR) | TSS will follow | NA | NA | NA | NA | NA |
| 3 | 37, F | 1.7 | Micro left, CSI- | DA | NR | 2.8 | Possible multifocality | Susp. active lesion left | 2.58 | Possible (TR) | TSS | No/  Negative | No | No | Improved | 1, from patient perspective |
| 5 | 38, F | 4.5 | Micro right^b^ | DA, TSS | >1 y | 2.4 | Possible multifocality, CSI+ | Active lesion anterolateral left, CSI+ | 0.98 | Unlikely (TR) | None, surveillance | NA | NA | NA | NA | NA |
| 13 | 59, M | 794.9 | Macro, possible CSI | DA | >1 y | 4.9 | Possible multifocality: cystic degenerated macro, invasion of clivus, sphenoid sinus and bone, ala major, CSI+ | Multiple active lesions alongside of necrotic lesion and in clivus | 1.01 | Possible (debulking) | TSS | Yes^c^ / PRL | No | No | Improved | 1 |
| 12 | 35, F | 7.9 | Hemorrhage, no adenoma | DA, TSS | NR | 3.4 | Micro dorsocaudal left | Active lesion left | 1.11 | Possible (TR) | None, surveillance | NA | NA | NA | NA | NA |
| 8 | 35, F | 4.1 | Micro left, CSI- | DA, TSS | <6 mo | 1.3 | Micro remnant lateral left, CSI- | Active lesion left, CSI- | 1.15 | Possible (TR) | TSS | Yes/ PRL | No | Yes | Improved | 1 |
| 15 | 30, M | 50.7 | Macro^b^ | DA, TSS | < 6 mo | 10.3 | Macro remnant posterior right, CSI+ | Susp. active lesion right between bifurcation Carotis, possible CSI | 1.07 | TSS possible | TSS (TR) | No/negative | No | No | Unchanged | 3 |
| **ID** | **Age (y), sex** | **Baseline** | | **Prior treatment** | | **At time of [^18^F]FET-PET/MRI^CR^** | | | | **MDT estimation of chance to achieve surgical goal** | **Treatment after [^18^F]FET-PET/MRI^CR^** | **Intraoperative findings correspond [^18^F]FET-PET/MRI^CR^/ IHC** | **complications** | **Biochemical remission** | **Clinical status** | **IOQ ^a^** |
|  |  | **PRL (xULN)** | **MRI** | **Type** | **DA Duration** | **PRL (xULN)** | **MRI** | **[^18^F]FET-PET/MRI^CR^** | **TBRmax** |  |  |  |  |  |  |  |
| 10 | 35, M | 69.6 | Macro, possible CSI | DA, 2x TSS | >1 y | 20.3 | Possible remnant | Diffusely increased activity | 1.03 | Repeat imaging advised | Repeat imaging | NA | NA | NA | NA | NA |
| 14 | 45, F | 6.5 | Micro central, CSI- | DA, TSS | NR | 11.4 | Macro right, possible CSI, RCC in pars intermedia | Active lesion right anterior | 1.32 | Likely (TR) | TSS | Yes/ PRL | Permanent: PTSD ^d^ | Yes ^e^ | Worse | 2 |
| 1 | 54, F | 1.4 | Micro left inferior, CSI- | DA | >1 y | 1.0 | possible micro left with possible CSI | Active lesion left with CSI | 1.35 | Possible (TR) | None, normalization after PRL menopause | NA | NA | NA | NA | NA |
| 18 | 39 F | 8.3 | Micro right, CSI- | DA | >1 y | 1.8 | Possible micro right | Active lesion right lateral | 0.96 | Likely (TR) | None, surveillance | NA | NA | NA | NA | NA |
| **Group 2: [^18^F]FET-PET/MRI^CR^ for radiological diagnosis** | | | | | | | | | | | | | | | | |
| 16 | 45, F | 15.0 | Macro ^b^ | DA, 2x TSS | >1 y | 8.3 | No certain adenoma | Susp. active lesion left, CSI+ | 1.04 | Unlikely (TR) | None, PRL halved after stopping gonadal replacement | NA | NA | NA | NA | NA |
| 17 | 35, F | NR | Macro right, CSI- | DA, TSS | >1 y | 2.4 | No certain adenoma | Susp. active lesion right against CS | 1.37 | Unlikely (TR) | started gonadal replacement | NA | NA | NA | NA | NA |
| 6 | 24, F | 3.5 | Macro ^b^ | DA,  TSS | >1 y | 2.1 | No certain adenoma | Active lesion right | 1.13 | Possible (TR) | TSS | Yes/ PRL, GH | Transient: sinusitis | Yes | Improved | 1 |
| 11 | 37, F | 3.3 | No certain adenoma | DA | >1 y | 3.6 | No certain adenoma | Active lesion right | 2.25 | Possible (TR) | TSS | Yes/ PRL, GH | No | Yes | Improved | 1 |
| **ID** | **Age (y), sex** | **Baseline** | | **Prior treatment** | | **At time of [^18^F]FET-PET/MRI^CR^** | | | | **MDT estimation of chance to achieve surgical goal** | **Treatment after [^18^F]FET-PET/MRI^CR^** | **Intraoperative findings correspond [^18^F]FET-PET/MRI^CR^/ IHC** | **complications** | **Biochemical remission** | **Clinical status** | **IOQ ^a^** |
|  |  | **PRL (xULN)** | **MRI** | **Type** | **DA Duration** | **PRL (xULN)** | **MRI** | **[^18^F]FET-PET/MRI^CR^** | **TBRmax** |  |  |  |  |  |  |  |
| 7 | 27, F | 6.3 | Micro, dorsocaudal left | DA | >1 y | 7.5 | No certain adenoma | negative | NA | Unlikely (TR) | Increase dose DA | NA | NA | NA | NA | NA |
| 9 | 49, F | 4.3 | micro mediolateral | DA | <6 mo | 10.8 | No certain adenoma | Negative | NA | Unlikely (TR) ^f^ | TSS | Chronic hypophysitis | No | Yes | Improved | 1 |

*CS* cavernous sinus, *CSI* cavernous sinus invasion, *DA* dopamine agonist, *F* female, *GH* growth hormone, *ID* study identification number, *IOQ* integrated outcome quadrants: *IOQ 1* – treatment goal achieved without permanent complications, *IOQ 2* – treatment goal achieved with permanent complication, *IOQ 3* – treatment goal not achieved, without permanent complications, *IOQ 4* – treatment goal not achieved with permanent complications, *IHC* immunohistochemistry, *M* male, *macro* macroadenoma, *micro* microadenoma, *mo* months, *NA* not applicable, *NR* not reported, *PRL* prolactin, *PTSD* post-traumatic stress disorder, *Q* quinagolide, *RCC* Rathkes cleft cyst*, TBRmax* maximum adenoma-to-background ratio, *TR* total resection, *TSS* transsphenoidal surgery, x*ULN times* upper limit or normal, *Y* year.

^a^ Integrated outcome quadrants as measured at 6 months postoperative

^b^ CSI unknown

^c^ Visibility was impaired due to large volume of blood loss from sphenoid sinus

^d^ Patient developed PTSD due to an event that occurred shortly before she was anesthetized

^e^ Patient was considered in biochemical remission with a prolactin 1.1xULN, as patient was using oral contraceptives during the measurement which can elevate prolactin levels

^f^ MDT agreed to surgical exploration despite an unlikely chance of achieving total resection due to incapacitating symptoms of galactorrhea, headaches and hypogonadism, and side effects of medication

**Supplementary Table 2** Description of case histories per patient

| **Group 1: [^18^F]FET-PET/MRI^CR^ for additional information** | | |
| --- | --- | --- |
| **Subcategory** | **Study ID** | **Case description** |
| Possible multifocality | 2 | Patient had no visible lesion on conventional MRI at diagnosis and was treated with quinagolide for approximately four years despite side effects (collapses with sinus arrest). After DA withdrawal hyperprolactinemia with hypogonadism reoccurred with a need for alternative treatment. Conventional MRI at time of [^18^F]FET-PET/MRI^CR^ raised the suspicion of a multifocal lesion, with a cystic lesion dorsal left and an adenomatous lesion on the bottom of the sella. [^18^F]FET-PET/MRI^CR^ confirmed uptake in the lesion left dorsal, without uptake in the cystic lesion. TSS was offered, with an estimated *likely* chance of total resection. The patient however postponed surgery due to personal reasons. |
|  | 3 | Conventional MRI at diagnosis showed a microadenoma left. Patient was treated with quinagolide for an unknown duration causing unacceptable side effects. Subsequent conventional MRI showed possible multifocality. [^18^F]FET-PET/MRI^CR^ performed to assess multifocality, confirmed an active lesion on the left. TSS was performed aiming for total resection, with a *possible* chance of remission and a low chance of complications. Intraoperatively no clear adenoma tissue could be identified. Histopathology consisted of minimal amorph tissue. Nevertheless, prolactin levels decreased to near-to-normal and the gonadal axis was restored, with a regular menstrual cycle. From a patient point of view, an IOQ 1 was achieved. |
|  | 5 | Conventional MRI at diagnosis showed a right sided small area of reduced enhancement. Patient was treated with cabergoline and bromocriptine for approximately two years and underwent TSS due to side effects, yielding positive PA. [^11^C]MET-PET/MRI^CR^ was performed in 2019 due to persistent disease, showing uptake left anterolateral in the sella, reaching in between the bifurcation of the internal carotid artery. After elaborate counselling, reTSS was postponed as the symptoms were mild (galactorrhea and mild psychological complaints, with regular menstrual cycle) and the chance of total resection was deemed limited due to CSI. In 2021, new irregularity of the patient’s menstrual cycle necessitated reevaluation of treatment options. Sequential Conventional MRIs raised the suspicion of multifocality. [^18^F]FET-PET/MRI^CR^ confirmed one left-sided lesion with CSI, corresponding with [^11^C]MET-PET/MRI^CR^. The MDT refrained from surgery due to an *unlikely* chance of total resection. Patient chose to continue biochemical surveillance as she experienced an acceptable quality of life without treatment. |
|  | 13 | Conventional MRI at diagnosis showed a macroadenoma with invasion of the sphenoid sinus, nasopharynx, right choana and nose septum to the contralateral side, with possible CSI. The patient was treated with cabergoline for approximately six years, during which he developed obsessive sexual preoccupations. Conventional MRI at that time showed a cystic degenerated macroadenoma in the clivus and sphenoid sinus with invasion of the ala major of the sphenoid bone and bilateral CSI. [^18^F]FET-PET/MRI^CR^ was performed to identify the location of the most active lesions and showed multiple active lesions alongside of a necrotic lesion and in the clivus. The patient underwent TSS aiming to reduce DA dose, as the need for intervention was high due to the obsessive-compulsive disorder. The chance of achieving this goal was estimated to be *possible*, with a moderate chance of complications. Debulking was limited by blood loss from the sphenoid sinus (600cc), decreasing visibility. Immunohistochemistry was positive and serum prolactin halved (to 2.9xULN) for which cabergoline was restarted in a lower dose (IOQ 1). However, the obsessive sexual preoccupation persisted, also in the context of autism, with an uncertain effect of DA treatment. |
| Remnant after TSS | 12 | The patient presented with a pituitary hemorrhage without a visible adenoma on conventional MRI at diagnosis. She was treated with cabergoline and quinagolide, which she took irregularly due to side effects. A previous TSS, which she underwent due to DA side effects, yielded positive immunohistochemistry and induced normalization of prolactin levels for two years, after which prolactin levels slowly increased. Conventional MRI was suspicious of a microadenoma remnant dorsocaudal left. [^18^F]FET-PET/MRI^CR^, performed to assess the exact localization and extension of the lesion, corresponded with the lesion on MRI, and showed no CSI. After a process of shared decision-making the patient chose not to undergo surgery, despite a *possible* chance of total resection, as her complaints were manageable (headache). Surveillance was continued. |
|  | 8 | Conventional MRI at time of diagnosis showed a left sided microadenoma, for which she was treated with cabergoline for two months, after which she underwent TSS due to side effects. Immunohistochemistry was inconclusive (positive for all hormones). Prolactin was persistently mildly elevated, for which cabergoline was restarted. Recurrence of side effects necessitated reTSS and conventional MRI showed a remnant lesion left lateral in the sella with unclear extension. [^18^F]FET-PET/MRI^CR^ confirmed the lesion visualized by MRI, without suspicion of CSI. [^18^F]FET-PET/MRI^CR^ guided surgery was performed with a *possible* chance of total resection. Intraoperative findings corresponded with [^18^F]FET-PET/MRI^CR^ and immunohistochemistry was positive for prolactin, normalizing prolactin levels without complications (IOQ 1). Approximately 6 months later she became pregnant. |
|  | 15 | Conventional MRI at time of diagnosis showed a macroadenoma dorsal in the sella in close relation with the optic chiasm. He was treated with cabergoline and quinagolide, for approx. 5 months in total, after which he underwent TSS due to side effects. Immunohistochemistry was positive for a prolactinoma, however prolactin levels remained elevated. The indication setting MRI (not shown in manuscript) suggested a macroadenoma remnant in the right CS. [^18^F]FET-PET/MRI^CR^ was performed to visualize the exact location and extension of the remnant to assess surgical possibilities, as the patient had a strong preference for another surgical attempt above radiotherapy. Functional imaging showed active prolactinoma tissue right lateral between the bifurcation of the carotid with possible CSI, in accordance with conventional MRI. Due to high disease burden, a surgical attempt was made despite a *possible* chance of achieving total resection and an intermediate risk of complications. Intraoperatively no clear adenoma was found, and immunohistochemistry was negative. TSS was uncomplicated, however no biochemical, or clinical improvement was achieved (IOQ 3). The MDT proposed radiotherapy, but the patient was reluctant due to the risk of pituitary failure; he continued biochemical and radiological surveillance (prolactin remained approx. 16xULN). |
|  | 10 | Conventional MRI at time of diagnosis showed a partly necrotic pituitary macroadenoma, extending inferoposteriorly into the sphenoid sinus and clivus with possible CSI. He was treated with cabergoline, quinagolide and bromocriptine. Due to side effects on all DAs he underwent a TSS twice, with positive immunohistochemistry, yet without normalization of prolactin and with persisting symptoms. Conventional MRI after the second TSS showed uncertain residual tissue. [^18^F]FET-PET/MRI^CR^ was performed to identify the most active location(s) of residual tissue for future treatment (TSS or radiotherapy). [^18^F]FET-PET/MRI^CR^ performed ten weeks postoperative, showed bilateral petroclival uptake but was inconclusive due to interfering CS activity and diffuse moderately increased tyrosine activity in sphenoid mucosa. Nine months postoperative, [^11^C]MET-PET/MRI^CR^ was performed, identifying two lesions; petroclival right and left. In retrospect, the pattern corresponded with [^18^F]FET-PET/MRI^CR^ results, except for the uptake in the sphenoid sinus seen on [^18^F]FET-PET/MRI^CR^ (due to postoperative mucosal inflammation). The patient underwent a successful debulking. |
|  | 14 | Conventional MRI at diagnosis showed a microadenoma left posterior in the sella. Patient was treated with quinagolide, cabergoline and bromocriptine for an unknown period of time, experiencing side effects on all. Her symptoms necessitated alternative treatment and subsequent conventional MRI showed a right sided macroadenoma remnant with possible CSI and a stationary RCC in the pars intermedia. FETPET/MRI^CR^, performed to visualize possible CSI, showed focal tracer uptake in the location of the right sided lesion on conventional MRI without CSI and no uptake in the cystic lesion. She underwent TSS with an estimated *likely* chance of total resection, a possible chance of symptom resolution (headache and mood disturbances) and a moderate risk of complications (mainly DI). Intraoperative findings corresponded with [^18^F]FET-PET/MRI^CR^ and immunohistochemistry was confirmative, resulting in biochemical remission. However, she developed PTSD due to a stressful event right she was anesthetized, for which she needed treatment (IOQ 2). |
|  | 1 | Conventional MRI at diagnosis showed a microadenoma left anterior in the sella. The patient was treated with cabergoline for just over a year, after which she stopped due to side effects. Conventional MRI was suspicious for a microadenoma on the left with possible CSI. Patient experienced various symptoms (amenorrhea, fatigue, cognitive complaints) despite only mildly elevated prolactin levels (approx. 1.5xULN) and the MDT was uncertain if the symptoms were caused by hyperprolactinemia or menopause. [^18^F]FET-PET/MRI^CR^ was performed for more information about the (extent of) CSI and to assess the activity of the lesion, which confirmed an active lesion with CSI as seen on MRI. TSS was deemed feasible with a *possible* chance of total resection. However, she underwent no further treatment, as prolactin levels normalized after going into menopause. |
| Remnant after DA treatment | 18 | Conventional MRI at diagnosis showed a right sided microadenoma, for which she was treated with quinagolide for twelve years, with three failed withdrawal attempts. Subsequent conventional MRI showed a possible microadenoma on the right side. [^18^F]FET-PET/MRI^CR^ was performed to determine the exact location of the lesion after long-term DA treatment and confirmed an active lesion corresponding with conventional MRI. Surgery was deemed possible, with a *likely* chance of total resection. However the patient did not undergo TSS, as prolactin levels remained only mildly elevated (<2xULN) after DA withdrawal, with a regular menstrual cycle. The symptoms she experienced were concluded to more likely be caused by other comorbidity (tension type headache, migraine, irritable bowel syndrome and ankylosing spondylodiscitis). Biochemical surveillance was continued. |
| **Group 2: [^18^F]FET-PET/MRI^CR^ for radiological diagnosis** | | |
| After TSS | 16 | Conventional MRI at diagnosis showed a large macroadenoma (CSI unknown). She was treated with quinagolide for 1 month after which she underwent emergency TSS due to apoplexy (histopathology unknown). Postoperatively she was treated with cabergoline and underwent reTSS 12 years later due to persistent disease and DA side effects (positive immunohistochemistry). Prolactin levels remained elevated and conventional MRI used for indication setting (not shown in manuscript) indicated an area of reduced enhancement on the left, yet it was unclear whether this concerned the resection cavity or residual adenoma tissue. [^18^F]FET-PET/MRI^CR^ was performed to identify residual adenoma tissue for radiotherapy, which identified an active lesion in the CS left. The patient did not undergo further treatment as prolactin levels halved after stopping gonadal replacement therapy (to approximately 2.5xULN), however her quality of life remained impaired due to panhypopituitarism (excluding DI). Biochemical surveillance was continued. |
|  | 17 | Conventional MRI at diagnosis showed a right sided macroadenoma without CSI or chiasmal compression. Patient was treated with bromocriptine and cabergoline (>5 years). She underwent TSS due to DA side effects yielding immunohistochemistry positive for prolactin and growth hormone. Three months postoperative hyperprolactinemia reoccurred. Conventional MRI at that time could not identify a remnant. [^18^F]FET-PET/MRI^CR^ showed active tissue dorsomedial of the right CS. Total resection was deemed *unlikely* based on [^18^F]FET-PET/MRI^CR^ results and the fibrotic tissue structure encountered during the previous TSS. Due to DA intolerance, she did not restart medical treatment, however, she experienced improvement of depressive symptoms after starting gonadal replacement therapy (serum prolactin approximately 3.4xULN). Surveillance was continued. |
|  | 6 | Conventional MRI at diagnosis showed a macroprolactinoma with compression of the optic chiasma (CSI unknown). She was treated with cabergoline for 9 years, after which she underwent TSS (histopathology inconclusive). After initial curation, hyperprolactinemia reoccurred and DA side effects necessitated reTSS. Conventional MRI, however, was not able to identify a target. [^18^F]FET-PET/MRI^CR^ identified a mildly active intrasellar adenoma right. She underwent reTSS with a *possible* chance of total resection and a moderate risk of complications. Immunohistochemistry was confirmative (prolactin and growth hormone), and prolactin levels normalized with only a transient complication (sinusitis)(IOQ1). |
|  | 11 | Conventional MRI at diagnosis showed a dubious lesion left in the sella that was only visible on dynamic sequences. After treatment with cabergoline for approximately 1.5 years with a failed withdrawal attempt, there was an indication for TSS due to DA side effects and a high disease burden. Conventional MRI showed two dubious lesions on the dynamic sequences, but no clear adenoma. [^18^F]FET-PET/MRI^CR^ identified increased tracer uptake right dorsolateral in the sella. She underwent TSS with an estimated *possible* chance of total and low risk of complications. Intraoperative findings corresponded with [^18^F]FET-PET/MRI^CR^ and immunohistochemistry was confirmative (prolactin and growth hormone). Prolactin levels normalized and no complications occurred (IOQ1). |
| After DA treatment | 7 | Conventional MRI at diagnosis showed a microdenoma dorsocaudal left in the sella. The patient was treated with cabergoline for approximately 6 years with mild side effects and no curation. She therefore wished to undergo surgery; however, conventional MRI showed no clear lesion. [^18^F]FET-PET/MRI^CR^ performed to identify a target for TSS was negative despite prolactin levels 7.5xULN and DA withdrawal > 9 weeks prior to imaging. The MDT refrained from surgery based on the negative imaging. Prolactin levels normalized after a DA dose increase and side effects were acceptable. |
|  | 9 | Conventional MRI at diagnosis showed a hypointense area mediolateral in the sella, suspicious for a microadenoma. She took cabergoline and quinagolide a few times, however, was unable to continue therapy due to severe side effects (vomiting). Conventional MRI at time of [^18^F]FET-PET/MRI^CR^ could not identify a certain adenoma. In accordance with conventional MRI, [^18^F]FET-PET/MRI^CR^ was negative, despite of prolactin levels 10.8xULN and DA withdrawal >11 weeks prior to imaging. Restarting DA treatment was not feasible due to the severity of side effects and the disease burden was high (severe galactorrhea, headaches, and hypogonadism). After extensive weighing of risks and benefits (*unlikely* chance of remission, high need for alternative treatment, low risk of complications), a surgical exploration was planned. Intraoperatively, possible, yet not certain adenoma tissue was found on the bottom, left and right of sella. Histopathology showed signs of a chronic hypophysitis. Prolactin levels normalized, and symptoms faded (IOQ 1). |

*IOQ* integrated outcome quadrants: *IOQ 1* – treatment goal achieved without permanent complications, *IOQ 2* – treatment goal achieved with permanent complication, *IOQ 3* – treatment goal not achieved, without permanent complications, *IOQ 4* – treatment goal not achieved with permanent complications, *MDT* multidisciplinary team, *PTSD* posttraumatic stress disorder, *TR* total resection, *TSS* transsphenoidal surgery, x*ULN* times upper limit of normal.
